# Maternal Overnutrition in Beef Cattle: Effects on Fetal Programming, Metabolic Health, and Postnatal Outcomes

**DOI:** 10.3390/biology14060645

**Published:** 2025-06-02

**Authors:** Borhan Shokrollahi, Myungsun Park, Gi-Suk Jang, Shil Jin, Sung-Jin Moon, Kyung-Hwan Um, Sun-Sik Jang, Youl-Chang Baek

**Affiliations:** Hanwoo Research Center, National Institute of Animal Science, Pyeongchang 25340, Republic of Korea; borhansh@gmail.com (B.S.); sunnypark411@korea.kr (M.P.); shinnanda16@naver.com (G.-S.J.); jins21@korea.kr (S.J.); moonsj27@korea.kr (S.-J.M.); umkh9969@korea.kr (K.-H.U.)

**Keywords:** maternal nutrition, fetal programming, muscle development, adipogenesis, beef cattle production

## Abstract

Maternal overnutrition and targeted supplements during pregnancy strongly affect fetal development in beef cattle, influencing gene expression, tissue development, and productivity after birth. As modern feeding practices often result in cows receiving energy and protein above requirements, understanding the balance between adequate nutrition and overconditioning is critical for sustainable beef production. This review synthesizes findings from recent studies on maternal overnutrition and supplementation, focusing on macronutrients (energy, protein, methionine) and key micronutrients (e.g., selenium, zinc). It evaluates the timing and impact of supplementation during different gestational stages, with emphasis on fetal muscle and adipose tissue development, immune function, and metabolic programming. The role of epigenetic mechanisms, such as DNA methylation and non-coding RNAs, is also discussed in relation to maternal dietary inputs. Mid-gestation supplementation promotes muscle growth by activating muscle-specific genes, whereas late-gestation diets enhance marbling and carcass traits. However, maternal overnutrition may impair mitochondrial efficiency, encourage fat deposition over muscle, and promote collagen synthesis, reducing meat tenderness. Recent evidence highlights sex-specific fetal programming differences, the significant impact of maternal diets on offspring gut microbiomes, and breed-specific nutritional responses, and multi-OMICs integration reveals metabolic reprogramming mechanisms. Targeted trace mineral and methionine supplementation enhance antioxidant capacity, immune function, and reproductive performance. Precision feeding strategies aligned with gestational requirements improve feed efficiency and minimize overfeeding risks. Early interventions, including protein and vitamin supplementation, optimize placental function and fetal development, supporting stronger postnatal growth, immunity, and fertility. Balancing nutritional adequacy without excessive feeding supports animal welfare, profitability, and sustainability in beef cattle systems.

## Simple Summary

Nutrition during pregnancy not only affects the health of cows but also plays a critical role in shaping the future of their calves. In beef cattle, what a cow eats while pregnant can influence the development of muscle, fat, and immune systems in her unborn calf. This review examines how cows with excess nutrients (overfeeding) or specific supplements such as protein, vitamins, or minerals during pregnancy impact the growth and health of their calves after birth. We explain how these feeding strategies can alter how genes are turned on or off before birth, which may lead to better meat quality or healthier animals. However, overfeeding can sometimes lead to unwanted effects like too much fat or lower meat tenderness. Recent research also highlights important differences between male and female calves, impacts on the calf gut microbiome, breed-specific responses, and advanced molecular analyses that can improve nutritional strategies. Feeding the right nutrients at the right time can help farmers raise stronger, healthier, and more productive animals, benefiting farmers, scientists, and anyone interested in animal welfare and sustainable meat production.

## 1. Introduction

Maternal nutrition during gestation has a strong effect on fetal development and subsequent performance in beef cattle [1,2]. Adequate nutrient supply is vital for developing key physiological systems in the fetus such as muscle growth, adipogenesis, immune responsiveness, and metabolic regulation in the developing fetus [3,4,5,6]. These lasting phenotypic effects are encapsulated by the concept of fetal programming, wherein the maternal environment drives enduring structural and functional changes in offspring [7,8]. Such effects often arise through epigenetic mechanisms including DNA methylation, histone modifications, and non-coding RNA regulation, which mediate transcriptional and metabolic pathways implicated in lifelong productivity [9,10].

Historically, research attention in ruminant systems has focused predominantly on maternal undernutrition and its detrimental consequences for progeny [4,11,12]. However, contemporary beef production practices, characterized by generous dietary planes or ad libitum feeding of energy- and protein-rich rations, have introduced a contrasting but equally problematic scenario: MO [13,14]. Under these high-intake conditions, pregnant cows often exceed optimal body condition scores, leading to impaired placental function, disturbed nutrient partitioning, and potential metabolic disorders that can negatively impact calf growth and carcass quality [15,16]. Therefore, managing MO is essential for maintaining productivity, as excessive in utero nutrient exposure may predispose offspring to unwanted fat deposition, reduced meat tenderness, and suboptimal feed efficiency [13,14].

Advances in precision feeding aim to match nutrient intake with pregnancy needs to avoid deficiencies or excesses [17,18]. Indeed, research suggests that carefully timed and moderate supplementation can improve outcomes such as fetal muscle fiber formation, marbling, and immune capacity [6,19]. Nonetheless, if nutrition during pregnancy is too low or too high, it may induce irreversible developmental changes that impair postnatal growth and carcass traits [3,4,20]. Recent research has shown how maternal diets, especially those with excess nutrients, alter fetal gene expression, impacting muscle growth, fat development, and lifelong metabolic health [21]. While certain micronutrients (e.g., zinc, Se, copper) also affect immune function and oxidative balance [22,23,24], the main concern in MO situations is exceeding energy or protein requirements, which promotes overconditioning in pregnant cows.

In this context, the present review focuses on MO in beef cattle, synthesizing current knowledge on how excessive dietary intake during gestation shapes fetal programming, affects metabolic health, and ultimately influences offspring growth, carcass characteristics, and overall performance. Understanding these mechanisms helps producers improve feeding strategies to avoid overnutrition and apply targeted interventions for more sustainable and profitable beef production.

## 2. Fetal Development Under MO

MO in beef cattle influences fetal development through alterations in placental function, critical windows of organogenesis, and key epigenetic mechanisms governing tissue differentiation. These processes have been well studied under maternal nutrient restriction. However, recent evidence shows that excess energy or protein intake can also disrupt normal fetal programming [2,4,14]. This section discusses the critical periods during which fetal tissues are most susceptible to overfeeding, explores how the placenta adapts (or fails to adapt) to excess maternal nutrients, and examines implications for muscle growth and long-term offspring performance.

### 2.1. Critical Windows in Fetal Development

Embryonic and fetal stages, from early embryogenesis to late gestation, are critical for forming cellular, metabolic, hormonal, and organ systems in beef cattle [11]. During these stages, skeletal muscle, adipose tissue, immune organs, and the placenta develop. Each of these tissues is highly sensitive to maternal nutritional imbalances, whether due to undernutrition or MO [2,11]. The principle of developmental programming posits that maternal conditions during pregnancy induce permanent structural and functional changes in offspring tissues, exerting lifelong effects on metabolism, growth, and health [2,11].

**Figure 1:**
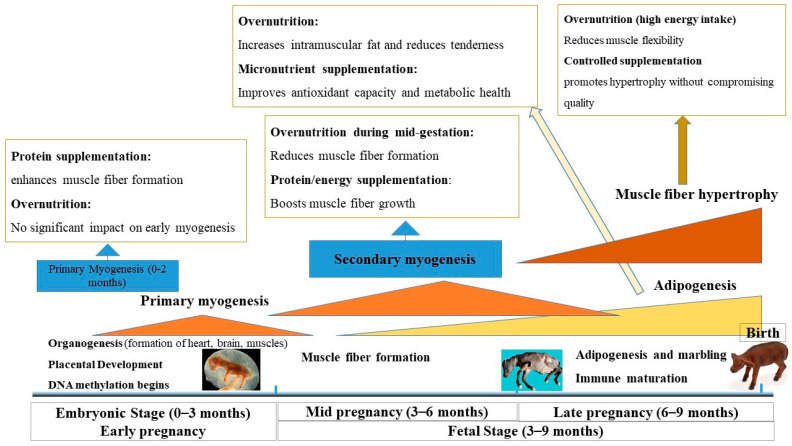
Critical windows of fetal development and the impact of maternal nutrition during pregnancy. This timeline illustrates key stages of embryonic and fetal development, highlighting periods of primary and secondary myogenesis, adipogenesis, and muscle fiber hypertrophy. MO and supplementation at different stages affect muscle and fat development. Early protein supplementation enhances muscle fiber formation, while MO in late gestation promotes intramuscular fat deposition and marbling. Controlled supplementation ensures optimal growth, balancing muscle development and fat accumulation.

The timing of organ and tissue formation is critical for fetal development. During the first trimester, organogenesis encompasses the formation of vital organs such as the heart, brain, liver, and muscles [25]. By around 50 days post-conception (dpc), organogenesis is largely complete [26,27]. After organogenesis, fetal development involves rapid growth and depends heavily on placental nutrient delivery [28]. If maternal nutrition is inadequate or excessive during this stage, it can harm placental function, causing restricted or excessive fetal growth, both affecting long-term metabolism and growth (Figure 1) [29,30].

### 2.2. Importance of Placental Development

The placenta is pivotal in mediating fetal nutrient and oxygen supply, directly influencing fetal growth outcomes [31,32,33]. When maternal nutrition is compromised—whether by deficiency or overfeeding—placental vascularization and nutrient transport efficiency may be disturbed [8,34,35]. In cases of undernutrition, inadequate placental development often results in IUGR and lower birth weights [8,34,35]. Conversely, MO can also disrupt normal placental function through altered metabolic environments and endocrine signaling [36].

The placenta may sometimes compensate for nutrient shortfalls by adjusting its structure and vascular function [37]. However, these adaptations may fail if maternal diets remain unbalanced for prolonged periods. Severe or prolonged nutrient restriction leads to impaired organogenesis and diminished muscle fiber formation [3,8]. At the same time, excess maternal nutrition may boost placental nutrient transfer beyond fetal needs. This can lead to fat accumulation in organs like the heart and muscle, which may reduce carcass quality and increase the risk of metabolic disorders later in life [38,39].

### 2.3. Nutritional Programming and Muscle Development

Muscle development in beef cattle primarily occurs in utero, underscoring the significance of maternal diet for lifelong growth and performance [40]. Mid-gestation (second trimester) is particularly critical for myogenesis, during which primary and secondary muscle fibers form [3,40]. Nutritional restriction in this window can irreversibly reduce the number of muscle fibers, thus limiting skeletal muscle growth potential [3,4] (Figure 1). On the other hand, MO may increase intramuscular fat deposition, potentially improving tenderness, flavor, and juiciness. However, this advantage may raise consumer health concerns if it also alters the fatty acid profile unfavorably [41].

Evidence suggests that optimal maternal nutrition during late gestation supports higher birth weights, enhanced muscle fiber hyperplasia, and increased adipocyte formation, which collectively bolster postnatal growth and meat quality [4,42]. Specific interventions, such as methionine supplementation, can induce epigenetic modifications (e.g., DNA methylation, alternative splicing). These changes improve muscle glycolysis, contraction efficiency, and cytoskeletal organization, key traits for feed efficiency and meat quality (Figure 1) [42,43,44]. In contrast, nutrient restriction during gestation compromises fetal muscle fiber counts, ultimately diminishing growth performance and feed efficiency in calves [45,46].

Recent research has shown that maternal feeding strategies, ranging from no protein–energy supplementation to full supplementation, differently shape nitrogen metabolism and protein retention. These differences influence offspring growth trajectories [47].

While calves can partially regain muscle mass after birth, the reduction in muscle fiber number due to mid-gestation nutrient restriction cannot be fully reversed [3,40,48]. Therefore, maintaining balanced maternal nutrition, avoiding both under- and overfeeding, is essential for optimal muscle development and robust postnatal performance in beef cattle [4,40,45].

### 2.4. Developmental Programming and Long-Term Growth

The developmental origins of health and disease framework posits that prenatal conditions, particularly maternal nutrition, have enduring effects on offspring growth, health, and overall productivity. The concept of fetal programming, introduced by Barker [7], underlines that poor gestational nutrition, whether deficient or excessive, can cause permanent changes in offspring metabolism, organ function, and disease susceptibility. In beef cattle, fetal programming is most evident in postnatal growth and carcass traits. Nutrient imbalances during key gestational periods often result in reduced growth, lower carcass quality, and impaired reproduction [8,49].

Although maternal undernutrition has historically received greater attention [3,4,42], growing evidence suggests that MO also disrupts normal developmental pathways, potentially leading to increased birth weight, disproportionate fat deposition, and decreased feed efficiency in offspring [50,51]. Studies consistently show that nutrient restriction during early to mid-gestation reduces fetal muscle fiber formation, slows growth rates, and impairs carcass quality [3,52]. These deficits are irreversible because muscle fiber numbers are set prenatally and cannot be increased after birth [3,40]. Moreover, placental function can be compromised by nutrient shortfalls, reducing fetal nutrient transfer [8,34]. Conversely, excessive maternal nutrient intake may enhance placental transport beyond fetal needs. This can lead to greater fat accumulation in the fetus and raise the risk of postnatal metabolic issues [13,14].

Research on strategic protein–energy supplementation underscores the critical role of gestational timing in shaping offspring performance. Late-gestation supplementation improves amino acid metabolism in both dams and fetuses. It elevates the plasma levels of taurine, glutamic acid, and histidine, key metabolites for fetal muscle development, metabolic programming, and postnatal growth efficiency [6,53,54,55]. Furthermore, this supplementation modulates pathways tied to histidine and beta-alanine metabolism, implying long-term benefits for feed efficiency and carcass attributes [55]. Polizel, et al. [56] further support these findings by demonstrating persistent changes in plasma metabolome profiles. These changes, especially in arginine biosynthesis and histidine metabolism, appeared during the rearing and finishing phases, reinforcing the idea that prenatal nutrition strategies can have lasting effects.

Likewise, mineral supplementation, especially Se and other trace minerals, facilitates robust immune function and uterine health during mid-to-late gestation. Organic Se supplementation, in particular, can reinforce immune responsiveness and reproductive success [57]. Nevertheless, the timing of such interventions remains pivotal: nutrient deficits early in gestation can induce excessive fat deposition, lower muscle mass, and diminish growth efficiency [58]. Similar caution applies when excess maternal nutrients are supplied early in pregnancy, as they may set the stage for offspring adiposity and associated metabolic drawbacks [13,14].

## 3. Effect of Maternal Feed Supplementation on Maternal Physiology

### 3.1. Impact on Body Weight, Body Condition, and Energy Balance

Maternal feed supplementation during pregnancy significantly influences BW and BCS, especially in the final trimester [8,13,14]. For instance, protein supplementation helps maintain BCS around 5.1, preventing declines seen in non-supplemented cows. It can also increase maternal BW at weaning while enhancing cyclicity at the start of fixed-time artificial insemination protocols [59]. Diets with 33% CP, 150% of requirement feeding, or corn supplementation at 0.2% BW have been shown to improve BW and BCS. These strategies enhance energy balance, reduce fat mobilization, and support better nutrient partitioning [53,60,61,62].

Mid-gestation protein supplementation (3.5 g/kg BW) can enhance BCS, weight gain, and DMI. It also improves digestibility and microbial protein synthesis, contributing to improved placental blood flow [53,63,64]. Sustained protein–energy supplementation throughout gestation may increase BW by 25–30 kg compared to mineral-only diets. This highlights the need for sufficient nutrient provision to preserve maternal energy reserves and support reproductive success [65]. A plant-based protein supplement given periconception has not consistently altered BW or BCS [64]. However, offspring from RPM dams show better feed efficiency and post-weaning growth, suggesting long-term metabolic benefits [66].

However, supplementation results are not always consistent across reproductive metrics. Some trials report no marked difference in pregnancy rates, even when BCS and weaning weights improve [59,67]. Overfeeding in the third trimester (100% or 150% of requirements) enhances BW and BCS at calving. However, it does not always translate into higher pregnancy rates [68,69]. RUP feeding during late gestation boosts ADG and final BW, but it does not clearly enhance fertility [54]. Micronutrient supplementation (e.g., copper, Se, zinc) may not significantly affect BW or BCS. However, it can improve mineral status and enhance placental nutrient transfer [70]. Fat supplementation strategies, such as high-fat bakery waste or specific oils, have also been used to maintain maternal weight and BCS through calving [71,72].

Energy balance is central to these outcomes. Severe nutrient restriction between day 30 and 190 of gestation reduces BW, BCS, and plasma glucose concentrations [73,74,75], while moderately overfeeding (125% total digestible nutrients, TDN) allows cows to gain weight without necessarily altering postpartum BCS [73,74,75]. MO at 120% ME decreases maternal fat mobilization pre-calving and stabilizes BCS, potentially aiding postpartum recovery [14]. HFAT supplementation may also improve fertility by increasing the number of cows that calve within the first 21 days of the breeding season [71]. Overall, balancing energy intake to align with fetal demands and avoiding extremes of under- or overfeeding remains vital for sustained maternal health and optimal reproductive outcomes.

### 3.2. Effects on Reproductive Function and Metabolic Parameters

While the goal of maternal supplementation is to enhance reproductive performance, its effect on actual fertility outcomes can be complex. Some studies report that protein supplementation elevates BCS and BW but does not significantly improve pregnancy rates relative to controls [59,76]. Similar patterns hold for enhanced follicular growth or cyclicity; these physiological indicators do not always culminate in higher conception rates [76,77]. Periconception RPM supplementation likewise did not improve pregnancy outcomes [66]. One recent report found that continuous protein–energy feeding (0.3% BW) elevated BW and rump fat thickness but conferred minimal benefits in reproductive traits, likely due to compensatory growth that offset early advantages [78].

Although better BCS and metabolism aid postpartum recovery, too much protein may impair pregnancy outcomes. This occurs due to ammonia and homocysteine toxicity, disrupted uteroplacental flow, and lower progesterone levels [79]. This underlines the importance of balancing rather than simply maximizing protein supplementation.

Nevertheless, specific interventions during late gestation do appear beneficial. Cows receiving 100% or 150% of requirements often show quicker follicular recovery and increased ovulation rates by 21 days postpartum, with some improvements in subsequent breeding seasons, particularly at the higher supplementation rate [68,80]. Even if reproductive outcomes do not improve immediately, these interventions can stabilize metabolism and improve nutrient use. For instance, RUP-based diets improve maternal protein synthesis and energy metabolism, whereas control diets yield higher methionine levels that may favor antioxidant capacity [54]. In addition, supplemented cows generally exhibit more stable glucose regulation and lower lipolysis (as indicated by reduced NEFA and triglyceride levels), minimizing the excessive mobilization of maternal reserves [61,81].

Similarly, vitamin and mineral supplementation early in gestation can augment amino acid concentrations in fetal fluids, enhancing nutrient transport [82]. A balanced maternal energy status throughout pregnancy provides a more favorable metabolic environment for both dam and fetus [83]. While these enhancements in metabolic indicators do not invariably translate into higher pregnancy percentages, they reinforce dams’ overall physiological resilience, a critical factor for subsequent conception and calving intervals.

### 3.3. Long-Term Economic and Production Benefits

In addition to physiological and performance improvements, maternal supplementation can yield economic advantages by increasing weaning weights, enhancing reproduction, and ultimately boosting farm profitability. For instance, cows fed 150% of their nutritional requirements during the third trimester produced heavier calves. This results in higher sale values and greater returns across subsequent reproductive seasons [68,80]. However, the net benefit depends on feed costs, calf prices, and breed-specific factors. For example, in Hanwoo cows, no significant performance differences were found between calves from supplemented and control dams [61].

Producers must weigh feed expenses and logistical efforts against potential returns like heavier weaning weights, shorter postpartum intervals, or improved cow longevity. Supplementation can reduce postpartum anestrus and lower the number of open cows, improving herd efficiency [84]. However, if it only sustains overconditioned cows without boosting fertility or calf performance, returns may be limited. This highlights the value of precision feeding, carefully aligning rations with gestational needs to control costs and improve reproductive and growth outcomes.

In situations where supplementation does elevate weaning weights and maternal BCS, producers may offset higher feed expenditures through increased income at sale [85]. Partial budget analyses that consider feed costs, labor, and market trends help assess whether gains in calf weight or fertility justify the added cost. If feed costs are moderate or if heavier calves command a premium, supplementation often delivers a favorable return on investment [85,86].

Post-calving management also affects profitability. For example, cows grazing in subirrigated meadows show better BW and BCS than those fed hay, resulting in heavier weaning weights [59]. Similarly, early-life strategies like creep feeding can enhance the benefits of maternal supplementation. Creep-fed heifers may initially exhibit higher body weight and backfat, although post-weaning growth often levels out. However, they tend to reach first-service pregnancy earlier, reducing long-term costs by lowering the number of open cows [87].

When choosing supplementation strategies, producers should weigh feed costs against potential gains in fertility, reduced postpartum anestrus, and heavier market calves [88]. When well-timed nutrient support ensures good maternal BCS, higher productivity, and fewer culls, the investment is often justified. But if overfeeding fails to produce matching gains in offspring, costs may exceed revenue [89]. Aligning gestational nutrition with postnatal management is essential for achieving productivity and profitability. This reinforces the role of precision feeding and cost–benefit analysis in modern beef systems.

## 4. Epigenetics and Nutritional Interventions

### 4.1. Epigenetic Mechanisms and Metabolic Imprinting

Epigenetics refers to heritable modifications in gene expression that do not alter the DNA sequence itself [90]. These changes including DNA methylation, histone modifications, and non-coding RNAs regulate gene activity and influence offspring growth, metabolism, and health. In beef cattle, metabolic imprinting denotes early-life nutritional interventions that induce enduring changes in muscle and adipose development, ultimately impacting carcass quality and reproductive traits [91,92].

#### 4.1.1. DNA Methylation

DNA methylation typically involves the addition or removal of methyl groups at cytosine–phosphate–guanine (CpG) sites, thereby modulating transcription. MO can disrupt methylation patterns in fetal tissues, affecting key genes involved in myogenesis, adipogenesis, and insulin signaling [13,52].

In some cases, the hypermethylation of muscle-regulatory genes may suppress myofiber formation, whereas hypomethylation in adipogenic genes can elevate intramuscular fat [41,93].

Early-weaned calves on high-concentrate diets also display specific DNA methylation changes that reinforce marbling [94]. These epigenetic adaptations persist into the finishing phase, underscoring the importance of balanced maternal and early-life nutrition [21,95]. Importantly, DNA methylation changes are not limited to muscle or adipose tissue. For example, restricting maternal nutrient intake (60% requirements) in Wagyu cattle significantly altered fetal thymus methylation, impairing immune signaling pathways (Ras, MAPK, cAMP) [96]. Maternal methionine supplementation in early gestation altered over 28,000 cytosine methylation sites in fetal and postnatal muscle. These changes affected genes related to contraction, PI3K signaling, mitochondrial function, and ROS homeostasis [21].

A recent multi-omics study found that small differences in maternal weight gain (0.28 vs. 0.79 kg/day) during early gestation changed fetal liver DNA methylation in beef calves. This affected key metabolic pathways such as lipid metabolism, Wnt signaling, and PI3K-Akt signaling [97]. These changes occurred in parallel with shifts in miRNA profiles, particularly the upregulation of miR-206, suggesting the coordinated epigenetic programming of hepatic development.

Taken together, these findings illustrate how maternal nutrition reshapes DNA methylation profiles across multiple organ systems, with long-lasting consequences for growth and health.

#### 4.1.2. Histone Modifications

Histone proteins organize DNA; chemical marks (e.g., acetylation, methylation, phosphorylation) on histone tails remodel chromatin structure. Open chromatin typically promotes gene transcription, whereas condensed chromatin restricts it. High maternal energy intake may cause histone modifications in fetal precursors, promoting fat cell formation over muscle development [13,19,93].

Direct bovine data are still emerging, but parallel research in sheep and rodents demonstrates that excess maternal nutrients elevate histone marks on adipogenic genes such as PPARG and C/EBPα, while downregulating those tied to muscle growth [98,99,100]. This histone-driven epigenetic reprogramming likely explains why offspring from overfed dams exhibit higher intramuscular fat but fewer muscle fibers.

#### 4.1.3. Non-Coding RNAs (miRNAs and lncRNAs)

ncRNAs, including miRNAs and lncRNAs, operate post-transcriptionally, shaping metabolic and developmental pathways [92]. MO can alter circulating miRNA profiles, which can affect fetal cell proliferation and growth [101,102]. For example, the upregulation of pro-adipogenic miRNAs (e.g., miR-103, miR-143) boosts adipocyte formation, while the downregulation of muscle-specific miRNAs hinders myogenesis [103].

Recent work also highlights the role of lncRNAs in mediating prenatal programming. In a 2 × 2 factorial study with Angus-cross heifers, vitamin/mineral-supplemented diets (at 110% requirements) and different target weight gains significantly changed fetal liver lncRNA expression at day 83, regulating fatty acid oxidation, glycolysis, amino acid metabolism, and mineral transport [92]. Another study found that prenatal protein–energy supplementation changed over 1800 lncRNAs in the muscle tissue of offspring aged 15–22 months. These were linked to Wnt signaling, angiogenesis, and adipocyte development [104]. These observations reinforce the notion that maternal diet and especially overfeeding can durably shape postnatal muscle and metabolic phenotypes via lncRNA-based regulation.

### 4.2. Transgenerational and Multigenerational Epigenetic Effects

Epigenetic modifications including DNA methylation, histone modifications, and non-coding RNAs can be transmitted across generations, influencing traits such as growth, feed efficiency, and reproductive capacity [10]. These are classified as multigenerational, when both the fetus (F1) and its germ cells (F2) are directly exposed during gestation, and transgenerational, where changes appear in F3 or later generations without direct exposure [105,106].

Recent work in beef cattle indicates the potential for these transgenerational effects. A multigenerational Zebu study found that good maternal conditions in previous generations improved F3 offspring traits. Reproductive traits were most affected, with up to 52.9% of variation explained by historical maternal environment [106]. Nutritional interventions during pregnancy may thus alter DNA methylation patterns in fetal tissues, and in some cases, these changes persist for multiple generations [107]. Although data in cattle are still emerging, findings in other livestock suggest that strategic gestational nutrition could help improve long-term productivity through epigenetic mechanisms.

miRNAs further mediate these heritable processes, regulating mRNAs essential for muscle development, fat deposition, and overall growth [9,108]. MO frequently upregulates pro-adipogenic miRNAs, elevating fat accrual, while nutrient restriction can downregulate miRNAs that favor muscle development [94,101]. By continuously shaping gene expression, these small RNAs may help transmit metabolic or compositional traits across generations.

While maternal influences dominate most epigenetic literature, paternal diet is increasingly recognized. In sheep, F0 rams supplemented with methionine from weaning to puberty exhibited persistent changes in sperm DNA methylation that were passed to F1 and F2 generations, affecting over 100 cytosines [109]. These epigenetic marks were associated with growth and reproductive phenotypes, such as altered scrotal circumference and loin muscle depth, in unexposed offspring [109]. Another study found that 216 differentially methylated regions and 824 differentially methylated cytosine sites emerged in F0 sperm following prepubertal dietary methionine supplementation, targeting genes implicated in puberty onset (e.g., DAZAP1, CHD7) and testis development (TAB1, MTMR2, CELSR1). As a result, F1 progeny had lower pubertal weight and smaller testes, highlighting paternal influence on epigenetic inheritance [110].

Collectively, these studies highlight that environmentally induced epimutations arising from either maternal or paternal nutritional exposure can escape traditional epigenetic “erasure” during gametogenesis, influencing traits across multiple generations. By combining nutritional strategies (e.g., avoiding MO or underfeeding), genetic selection, and an understanding of epigenetic inheritance, producers can optimize muscle growth, fat development, and reproductive traits in beef cattle [41]. MO can enhance marbling but also increase collagen, which reduces tenderness. Its timing and intensity must be managed carefully to maximize benefits and avoid negative effects.

## 5. Impact of MO on Fetal Gene Expression

The effects of MO on fetal gene expression are well-documented, but a mechanistic synthesis is still needed to understand how these prenatal changes affect postnatal physiology. MO during gestation alters fetal gene expression in skeletal muscle, adipose, connective tissue, and energy metabolism (Table 1). These changes shape growth potential, carcass traits, metabolic efficiency, and long-term health.

### 5.1. Impacts on Muscle Development

MO and nutritional interventions affect myogenesis by altering gene expression and epigenetic regulation during gestation. Mid-gestation protein supplementation affects more than 300 genes. It upregulates muscle markers (e.g., LOC107131843, KRT8/18/19) and downregulates mitochondrial genes (e.g., AK9, LOC510904, CEACAM19), shifting fetal energy use toward fatty acids [111,112]. Methionine supplementation changes DNA methylation and splicing, reducing MYF5 expression and altering histone modifiers like ASH1L and NSD1. These effects link one-carbon metabolism to muscle development [43,52,113,114,115].

In late gestation, higher protein intake upregulates p70S6k and GSK3B mRNA, promoting muscle growth without triggering catabolic markers (MuRF1, Atrogin-1) [53]. In contrast, excess energy intake downregulates oxidative and myogenic genes (e.g., PPARGC1A, THBS1), impairing myogenesis and mitochondrial function [116]. PUFA supplementation upregulates MYH7 and C/EBPβ in fetal muscle, improving lipid metabolism [117].

Overfeeding promotes glycolytic (type 2x) fibers and increases fat and collagen expression, even when MyoD and MyoG levels remain stable [13,118]. RPF supplementation in late pregnancy shifts the balance toward glycolytic fibers [119]. Mid-gestation protein–energy supplementation (e.g., soybean meal) enhances fetal muscle growth by upregulating IGF1 and IGF2 [120]. Se supplementation affects contractile genes like MYOG and MYH1. However, Se intake in early gestation may temporarily reduce their expression and slightly affect birth weight [121,122].

### 5.2. Regulation of Adipogenesis and Fat Deposition

**Figure 2:**
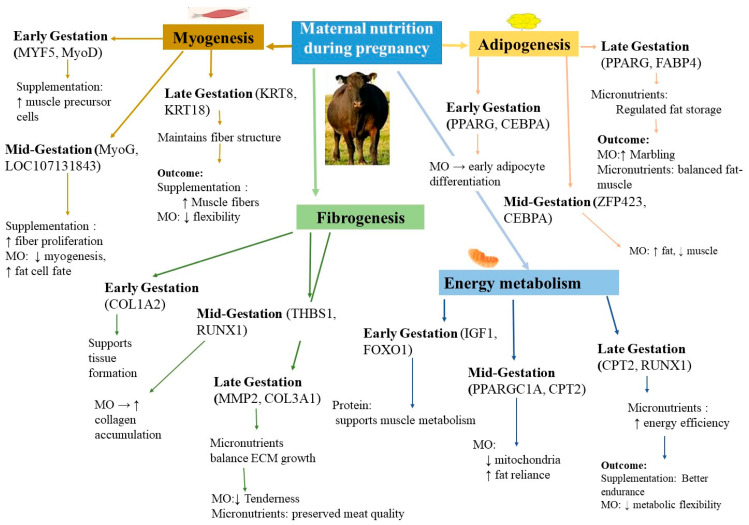
Maternal nutrition reprograms key fetal pathways. Gold (myogenesis), orange (adipogenesis), green (fibrogenesis), and blue (energy metabolism) arrows indicate major developmental axes. Upward (↑) and downward (↓) arrows show increased or decreased activity under protein/micronutrient supplementation or MO, respectively. Icons denote the maternal plane (cow), muscle fiber development (muscle icon), fat deposition (fat droplet), collagen deposition (collagen icon), and mitochondrial/energy pathways (mitochondrion). Abbreviations: MYF5 (myogenic factor 5), MyoD (myoblast determination protein 1), KRT8/18 (keratins 8 and 18), PPARG (peroxisome proliferator-activated receptor γ), FABP4 (fatty acid binding protein 4), ZFP423 (zinc finger protein 423), COL1A2/COL3A1 (collagen types I α2/III α1), THBS1 (thrombospondin 1), RUNX1 (runt-related transcription factor 1), MMP2 (matrix metalloproteinase 2), IGF1 (insulin-like growth factor 1), FOXO1 (forkhead box O1), PPARGC1A (PPAR γ coactivator 1 α), CPT2 (carnitine palmitoyltransferase 2).

**Figure 3:**
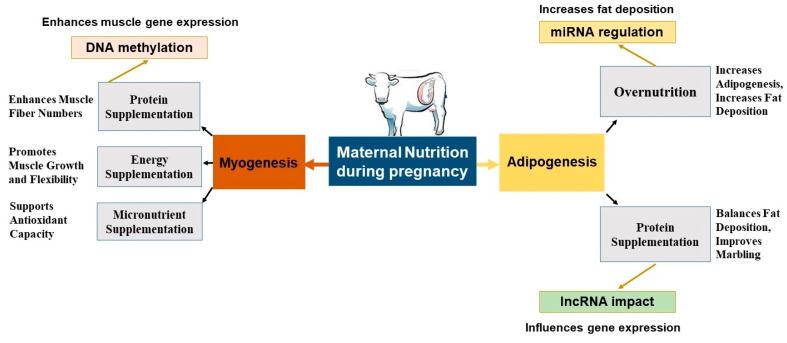
Maternal nutrition during pregnancy differentially programs myogenesis and adipogenesis. The blue central box denotes the maternal dietary plane. The orange pathway (**left**) traces myogenesis: protein, energy, and micronutrient supplementation increase muscle-fiber number, growth flexibility, and antioxidant capacity; epigenetic activation via DNA methylation and lncRNA networks further upregulates myogenic genes. The yellow pathway (**right**) traces adipogenesis: MO or targeted micronutrients upregulate adipocyte differentiation and fat deposition through miRNA regulation and lncRNA-mediated gene control; a balanced micronutrient supply can instead improve marbling while limiting excess fat. Arrow thickness indicates the direction of regulation: upward arrows = stimulation; downward arrows = repression. Abbreviations: MO, MO; lncRNA, long non-coding RNA; miRNA, microRNA.

Maternal nutrition during pregnancy influences fetal fat development by altering gene networks and epigenetic regulators that direct cell fate toward muscle or fat. Late-gestation PUFA supplementation upregulates MYH7 and C/EBPβ while repressing MYF5 in muscle precursors and decreases SCD in adipose tissue, indicative of leaner growth [117]. Protein–energy supplementation similarly promotes the expression of SPRING1 and FBXW8, increasing backfat thickness [65,123]. Conversely, MO elevates PPARG and ZFP423, tipping the balance toward adipogenesis at the expense of myogenesis (Figure 2). Fetuses from nutrient-restricted dams show higher UCP1 and PGC1α, indicating BAT development. Overfed dams produce fetuses with elevated LEP, CEBPA, and PPARG, typical of WAT [102]. Figure 3 summarizes how MO, micronutrient supplementation, and epigenetic mechanisms (e.g., miRNA regulation) influence fat accumulation and marbling.

Changes in miRNAs (e.g., miR-103, miR-107) and β-catenin downregulation promote adipocyte differentiation [3,13,94,124,125,126]. High RUP intake also increases ZFP423, PPARG, and C/EBPα, encouraging intramuscular fat deposition [54].

Specific nutrients fine-tune these effects. RPF lowers PPARα, promoting subcutaneous fat, while vitamin A activates RARβ, DLK1, and PPARG in intramuscular cells without affecting subcutaneous fat [119,127]. These gestational diets coordinate the balance of muscle and fat development, strongly affecting carcass quality, metabolic health, and production efficiency [128,129].

### 5.3. Fibrogenesis and Connective Tissue Formation

MO profoundly alters fetal ECM integrity by affecting collagen and adhesion gene networks. Overfeeding during mid-to-late gestation downregulates ECM regulators (THBS1, CD44, CNN1, JUND) and upregulates MMP2, a matrix-degrading enzyme. This shift may weaken muscle by disrupting cytoskeletal organization [116]. MO also enhances fibrogenesis by increasing the expression of COL3A1, FN1, and TGF-β, which promote collagen cross-linking and connective tissue stiffness [13,119,130]. Histological analyses corroborate these transcriptomic changes, revealing elevated type III collagen deposition in fetal muscle under excessive maternal nutrition [13,131,132]. Nutritional interventions can influence these outcomes. PUFA supplementation upregulates COL1A2, COL8A1, and FBN3 in muscle, reinforcing ECM structure [117]. Methionine supplementation reduces histone methylation and suppresses fibrogenic regulators like ASH1L and NSD1, limiting excess collagen synthesis [115]. In nutrient-restricted conditions, genes like PCDH19 are upregulated to stabilize the ECM [133,134]. In contrast, imbalanced diets increase COL1A1 and LOX in subcutaneous fat, connecting fat growth to fibrotic remodeling [54,120,131,135].

### 5.4. Energy Metabolism

MO or restriction alters fetal energy metabolism by reprogramming genes related to mitochondria, glucose use, and fat oxidation. Excess maternal energy often reduces PPARGC1A and other oxidative genes (e.g., THBS1, CD44, RUNX1). This shifts fetal muscle toward fatty acid oxidation instead of glucose use [116,121]. Our previous study showed that ADCY6 is upregulated, heightening ATP-to-cAMP conversion as an adaptive response to nutrient excess [116]. As shown in Figure 2, MO suppresses PPARGC1A, impairing mitochondrial function and reducing oxidative capacity in muscle tissues.

Nutritional supplements may either reduce or worsen these metabolic shifts. Methionine supplementation improves ATP production and redox status. It also lowers CBS expression, influencing one-carbon metabolism and the methylation of metabolic genes [100,113,115]. Moderate protein–energy supplementation increases PITPNA and CDK5R1, supporting mitochondrial growth and insulin signaling in fetal tissues [53,121]. Se activates PRKAG3, enhancing AMPK pathways that promote energy efficiency. However, early excess Se may suppress FOXO1 and FOXO3, showing how these pathways are sensitive to timing [121,132].

Maternal energy intake shapes fetal fat metabolism. Overfeeding raises FASN and CPT2, boosting fat production and oxidation [18,119,136]. In contrast, restriction increases FAS and PREF-1, pushing progenitors toward fat cell development [18,129]. Epigenetic mechanisms also control energy use. Methylation at PCDH19 and shifts in miRNAs (e.g., miR-15b, miR-27b) adjust the balance between oxidation and fat storage [102,134].

These gene and epigenetic changes reveal how maternal diets reprogram the fetus. They lead to lifelong effects on feed efficiency, metabolic health, and productivity.

### 5.5. Long-Term Consequences of MO-Induced Fetal Gene Expression

Although MO-induced transcriptional and epigenetic changes are well established [3], their true impact lies in how they influence the animal throughout life and even across generations [93]. First, deficits in primary and secondary myogenesis (e.g., reduced MYF5 and PPARGC1A activity) [129,137] limit muscle fiber number and alter fiber-type composition. This results in lower lean mass, slower growth, and reduced feed efficiency across the lifespan [3,13,93,116]. Second, the overactivation of adipogenic genes (PPARG, ZFP423, C/EBPβ) leads to more intramuscular fat and improved marbling [13,94]. However, it also increases collagen cross-linking (COL3A1, FN1), which can reduce meat tenderness and affect consumer preference [13,118,130]. Third, changes in mitochondrial (THBS1, RUNX1, ADCY6) and insulin-signaling genes (PITPNA, CDK5R1) create a metabolic profile with reduced oxidative capacity and disrupted glucose–lipid balance. These animals may have lower resilience to nutritional or environmental stress and a higher risk of metabolic disorders [98,121,134].

MO affects more than just growth and carcass traits. Changes in immune and endocrine gene networks, shaped by interventions like trace minerals and methionine, have been associated with differences in disease resistance, vaccine response, and reproductive milestones such as age at puberty and conception rates [65,138,139]. Emerging evidence shows that epigenetic marks such as DNA methylation, histone changes, and ncRNA profiles can persist into the F₂ and F₃ generations. These heritable changes affect growth, fertility, and carcass traits in animals that were never directly exposed to the original maternal nutrition [105,108].

These findings highlight that MO-induced fetal programming is not merely a short-term effect. It plays a major role in lifetime productivity, animal health, and performance across generations of beef cattle.

**Table 1:** Differential expression of genes (or regulatory RNAs) in bovine fetuses and postnatal calves in response to specific maternal nutritional interventions, with inferred functional roles and phenotypic outcomes.

Gene(s)	Function	Nutritional Status	Stage	Expression Change	Impact	References
**LOC107131843**	Insulin receptor signaling (Akt3-mTOR pathway)	Prot. Sup.	Postnatal	↑	Promotes muscle hypertrophy	[111]
**KRT8, KRT18, KRT19**	Apoptosis regulation and muscle fiber structure			↑	Balances protein synthesis and degradation, promoting muscle development	
**CLSTN3**	Cell adhesion			↑	Enhances muscle integrity	
**ANGPTL4**	Lipid metabolism			↑	Supports lipid metabolism and energy regulation	
**KCNH3**	Potassium ion transport			↑	Enhances muscle function	
**ENSBTAG00000055143**	Mitochondrial energy metabolism			↑	Improves energy efficiency and muscle development	
**AK9**	Nucleobase metabolism			↓	Reduces metabolic activity in muscle	
**LOC510904**	AA transport			↓	Limits nutrient transport	
**CEACAM19, ENSBTAG00000032057**	ATP synthesis and energy production			↓	Alters mitochondrial function	
**ALKBH1**	DNA methylation (N6-methyladenine regulation)			↑	Promotes muscle proliferation while reducing differentiation	
**MYF5**	Myogenic regulatory factor (muscle fiber formation)	Energy + Prot. Sup. PUFA/MPN	Postnatal	↓	Reduces muscle fiber differentiation. 2- PUFA supplementation may preserve muscle development by maintaining MYF5 expression	[94,115]
**MYH7**	Muscle fiber development	PUFA	Postnatal	↑	Increased muscle development from birth to weaning	[117]
**Zfp423**	Regulates early preadipocyte commitment	MO, PUFA, RUP	Fetal and postnatal	↑↓	Promotes adipogenesis and fat deposition via PPARγ activation	[13,54,117]
**PPARG**	Adipogenesis and lipid metabolism	MO, MPN, PUFA, RUP	Fetal and postnatal	↑-	Increases fat accumulation, marbling, and adipogenic programming no significant difference (PUFA)	[13,54,94,117,120,124,140]
**AGRP**	Appetite regulation (appetite-stimulating)	A high-protein or nutrient-rich diet	Postnatal	↑	Increases appetite and DMI	[141]
**C/EBPα**	Adipocyte differentiation and fat deposition	MO, MPN, PUFA, RUP, low-energy diet	Fetal and postnatal	↑	1. Increases adipogenesis and fat accumulation in muscle through interaction with PPARγ (MO). 2. Enhances adipogenic programming and fat deposition during fetal development (MPN).	[13,54,94,117,120,124,129]
**PSPH, PSAT1, PHGDH**	Enzymes in serine biosynthesis pathway	Met Sup.	Postnatal	↓	Reduced serine synthesis and AA metabolism	[115]
**SLC7A1, SLC7A5, SLC3A2**	AA transporters			↓	Limited AA transport and reduced mTOR signaling	
**CBS**	Enzyme in the transsulfuration pathway			↓	Decreased transsulfuration pathway; potential methionine remethylation	
**DNMT3a**	DNA methyltransferase (de novo methylation)	Energy + Prot. Sup.		↓	Possible effects on epigenetic regulation and gene expression	
**TGFBR2, SMAD2, SMAD4**	TGF-β signaling pathway			↓	Suppression of muscle growth, increased fibroblast activity	
**Wnt-related (e.g., FZD7, APC)**	Wnt/β-catenin pathway			↓	Reduced myogenesis, favoring adipogenesis	
**MTHFD2, ALDH1L2, MTHFD1L**	Mitochondrial folate metabolism			↓	Impacts one-carbon metabolism and methyl donor availability	
**β-catenin**	Suppresses adipogenesis (Wnt/β-catenin pathway)	MO/Met Sup.	Fetal and postnatal	↓	Favors adipocyte differentiation over myogenesis	[13,94,113]
**PCDH19**	Protocadherin; involved in immunity	Low maternal diet	Postnatal	↑	Regulates energy metabolism and growth, linked with feed efficiency and metabolic weight	[134]
**GSTM1/2**	Glutathione S-transferase; antioxidant activity	Prot. En. Sup.		↑	Regulates oxidative stress and detoxification	
CAST	Calpastatin; inhibits calpain enzymes	Prot. En. Sup		↑	Regulates muscle protein degradation, impacting meat quality	
**μ-Calpain**	Protein turnover and muscle fusion	HE diet	Fetal	↑	May indicate enhanced myoblast fusion and larger muscle fiber potential	[129]
**PAX3, GLI**	Essential for skeletal myogenesis	Met Sup.	Postnatal	↓	Reduces myocyte development	[113]
**TGF-β**	Regulates fibrogenesis and collagen deposition	MO, MPN, High-RUP diet	Fetal and postnatal	↑	Increases muscle fibrosis and collagen cross-linking	[13,54,94]
**COL3A1**	Collagen type III; fibrosis	MO	Fetal	↑	Promotes connective tissue formation and fibrosis	[13]
**FABP4**	Fatty acid metabolism	MPN	Postnatal	↑	Supports fat accumulation during fetal development	[117,124]
**FASN**	Fatty acid synthesis	MPN, PUFA	Fetal and postnatal	↑	Increases marbling in muscle	[94,117,124,140]
**IGF2R**	Insulin-like growth factor 2 receptor, regulates cell differentiation and apoptosis	HSD	Postnatal	↑	Facilitates IGF2 degradation to prevent fetal overgrowth	[95]
**MEG8**	Non-coding RNA associated with muscle growth			↑	Possibly involved in fetal programming and muscle development	
**DNMT3a**	DNA methyltransferase, involved in epigenetic regulation			↑	Modulates DNA methylation and epigenetic programming	
**PDGFRα**	Marker of fibro-adipogenic progenitor cells	High-RUP diet	Postnatal	↑	Enhances adipogenic and fibrogenic potential	[54]
**SCD**	Stearoyl-CoA desaturase, regulates fat composition	MPN, PUFA	Postnatal	↑↓	Increases (MPN) and decreases (PUFA) marbling in muscle	[94,117,124]
**PPARGC1A**	Mitochondrial biogenesis and energy metabolism	MO	Postnatal	↓	Reduces muscle oxidative capacity, suppresses energy metabolism	[116]
**miR-103, miR-107**	Regulate lipid metabolism (target CAV1)	MPN	Postnatal	↑	Modulate insulin sensitivity	[124]
**miR-27a/b, miR-130a**	Suppress adipogenesis (target PPARG, C/EBPα)			↓	Facilitate fat accumulation	
**miR-143**	Promotes adipocyte differentiation (target DLK1)			↑	Enhances adipogenesis	
**ADCY6**	Converts ATP to cAMP	MO	Postnatal	↑	Adapts energy metabolism	[116]
**COL1A1, COL1A2**	Collagen organization and cross-linking	MO/Met Sup.	Postnatal	↑	Promotes fibrosis and connective tissue development	[113,135]
**HSPA6, HSPA1A**	Cellular stress response	MO	Postnatal	↑	Protects cells from metabolic stress	[135]
**NME1, MAPK4**	Cell proliferation and differentiation	LPN	Postnatal	↓	Reduces muscle development	[94]
**SCUBE1, CARD14**	Immune signaling and inflammation			↑	Induces inflammatory response	
**PREF-1**	Inhibits adipocyte differentiation	HE maternal diet	Fetal	↑	Delays adipogenesis	[129]
**IGF-II**	Promotes muscle growth			Tendency ↑	Promotes fetal muscle development, though without observable fiber differences	
**AGR**	Appetite stimulation	Periconception (HPeri/LPost)	Postnatal	↑	Increases feed intake	[141]
**FGF2, PPARα**	Regulate muscle hypertrophy and metabolism	Mid-gestation supplementation	Postnatal	↑	Enhances muscle growth and energy metabolism	[120]
**MYF3, MYF6, MYH1, MYH3**	Muscle structure and contraction	Se Sup. (TR1/TR3)	Postnatal	↓ (TR1)/↑ (TR3)	Affects muscle development depending on trimester	[121]
**MYOG**	Myogenin; regulates muscle differentiation	Maternal PS/Se Sup., Low-energy diet	Fetal and postnatal	↑	Promotes muscle fiber differentiation and regulates hypertrophy and atrophy	[121,129,134]
**ABCA6, ABCB11, SLC27A6, SLC2A2, SLC2A4**	Nutrient transport and metabolism	Vit/Min Sup.	Fetal	↑	Enhances metabolic activity	[140]
**MT1A, MT1E, MT2A**	Metallothioneins involved in metal ion binding			↑	Enhances metal ion homeostasis	
**PPARD**	Regulates fatty acid oxidation			↓	Reduces fat oxidation	
**FADS2, LPL**	Lipid synthesis and metabolism			↓	Reduces fat storage	

↑/↓—up- or downregulation of transcript or protein abundance; AA—amino acid; AGR—agouti-related peptide; AKT3—RAC-α serine/threonine kinase 3; ALKBH1—AlkB homolog 1; APC—adenomatous polyposis coli; bta-miR-…—bovine micro-RNA; CAST—calpastatin; CPT2—carnitine-palmitoyl-transferase 2; DNMT3a—DNA-(cytosine-5)-methyl-transferase 3α; FABP4—fatty-acid-binding protein 4; FADS2—fatty-acid desaturase 2; FASN—fatty-acid synthase; FOXO1—forkhead box O1; GSTM—glutathione-S-transferase mu; IGF—insulin-like growth factor; KRT—keratin; lncRNA—long non-coding RNA; MAPK4—mitogen-activated protein kinase 4; MMP2—matrix metalloproteinase 2; MYF/MYH/MYOG—myogenic regulatory or myosin heavy-chain genes; PPARG/PPARGC1A/PPARD—PPAR γ, its co-activator 1α, and PPAR δ; PTPRC—protein-tyrosine-phosphatase receptor-type C; RUNX1—runt-related transcription factor 1; SCD—stearoyl-CoA desaturase; SMAD—mothers-against-decapentaplegic homologue; TGF-β/TGFBR2—transforming-growth-factor-β and its receptor; WNT/FZD7/β-catenin—canonical Wnt/β-catenin signaling components; ZFP423—zinc-finger protein 423; LPN—low plane of nutrition; MPN—medium plane; HPN—high plane; MO—maternal overnutrition; PUFA—poly-unsaturated fatty-acid supplementation; RUP—rumen-undegradable protein; Prot. Sup./Prot. En. Sup.—protein or protein-plus-energy supplementation; Met Sup.—rumen-protected methionine; Vit/Min Sup.—vitamin and/or mineral supplementation; Se Sup.—selenium supplementation (TR1 = first trimester, TR3 = third trimester); HSD/LHD—high- or low-starch diet; HPeri/LPost—high nutrition periconception, low post-conception.

## 6. Effect of Maternal Nutrition During Pregnancy on Postnatal Outcomes and Production Traits

Maternal nutrition during gestation strongly influences postnatal growth, health, and productivity in beef cattle. Both over- and underfeeding relative to recommended energy or protein requirements can cause lasting changes in growth, immunity, reproduction, carcass traits, and metabolic resilience. While earlier sections focused on in utero mechanisms like epigenetic and gene expression changes, this section emphasizes the observed phenotypic and performance outcomes.

### 6.1. Growth Performance and Development

Numerous studies demonstrate that maternal energy and protein intake significantly affect calf birth weights and subsequent growth. For instance, a high-energy (HE) diet during the final 45 days of gestation can boost birth weight and enhance neonatal immune and antioxidant capacity [138]. Similarly, Wilson et al. [75] reported that higher prepartum energy intake correlates with increased calf birth weights, underscoring the importance of energy-rich diets in late gestation. When RPM was provided near conception, offspring showed higher birth weights and improved postnatal ADG. This suggests that early gestational nutrition enhances growth efficiency [66]. These phenotypic outcomes are linked to gestational transcriptional changes. Reduced MYF5 and increased PPARG and ZFP423 expression shape muscle fiber development and fat cell formation.

In addition to late-gestation feeding, periconception and mid-gestation dietary strategies have yielded both transient and persistent effects on calf muscle development. Periconception protein supplementation increases the longissimus dorsi area at birth, likely through better nutrient partitioning toward muscles [141]. In contrast, Costa, et al. [142] found that mid-gestation protein restriction reduced offspring muscle fiber number, a deficit still evident at 450 days. However, changes in fiber-type composition (e.g., MHC2X) were observed only at 30 days and not maintained through finishing. Conversely, high-level MO during late gestation does not always yield long-term benefits. Several studies report no significant differences in birth, weaning, or carcass traits between supplemented and control groups [6,14,68,74,118]. Likewise, Lawson [69] reported that late-gestation protein or methionine changes had little impact on pre-weaning growth. In contrast, postnatal management (e.g., grazing vs. drylot) had a greater effect on calf performance and health. These mixed results suggest that the effectiveness of gestational nutritional programming depends heavily on when and how it is applied and on the conditions after birth.

Nevertheless, protein supplementation remains consistently beneficial. Heifer calves from supplemented dams exhibited higher 205-day weaning weights and body weights at both pre-breeding and pregnancy diagnosis, likely reflecting improved fetal muscle development from enhanced AA availability [12,143]. Cracco et al. [78] similarly observed only a trend toward heavier weaning weights in calves from fully supplemented dams, with no significant differences by 12–22 months of age. Similarly, Nascimento et al. [64] reported that mid-gestation protein supplementation increased birth and weaning weights, enhanced skeletal growth, and enlarged muscle fibers. These effects varied by sex.

Increasing maternal CP intake by 5% raised birth weights and improved ADG, ultimately increasing carcass weights and FCR in male progeny [144]. However, both low and excessively high protein intake can impair fetal muscle development and calf growth [79].

**Figure 4:**
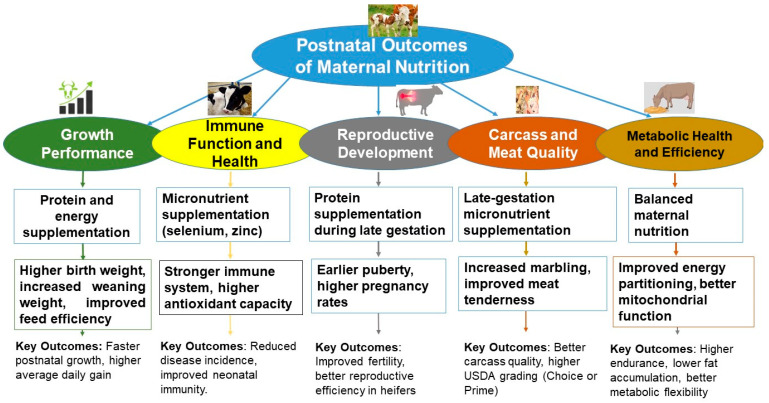
Postnatal outcomes linked to specific maternal nutrition strategies. The central blue ellipse denotes the overarching theme of Postnatal Outcomes of Maternal Nutrition. The five colored ovals highlight major performance domains: green = Growth Performance; yellow = Immune Function and Health; dark grey = Reproductive Development; orange = Carcass and Meat Quality; and brown = Metabolic Health and Efficiency. Under each domain, the grey rectangles list representative gestational interventions (e.g., protein-plus-energy, trace-mineral or micronutrient supplementation, balanced diets), and the white rectangles below summarize the principal offspring responses. Upward arrows indicate the direction of improvement. Key reported benefits include higher birth and weaning weights, stronger neonatal immunity, earlier puberty and improved fertility, superior marbling and tenderness (higher USDA grading), and enhanced energy partitioning with better mitochondrial efficiency. Abbreviation: USDA, United States Department of Agriculture.

Early- to mid-gestation supplementation can similarly impact growth. Cracco et al. [78] found that prenatal protein–energy supplementation increased weaning weights but had little effect on ribeye area or fat thickness. This suggests that compensatory growth may reduce long-term advantages. Similarly, Tanner et al. [62] reported that corn supplementation during mid-to-late gestation produced heavier calves at 21 days postpartum, although it did not affect birth or final weaning weights. Other studies have also reported a minimal impact of gestational supplementation on birth or weaning weight [59]. In another trial, LFAT supplementation outperformed high-fat or no-supplement strategies, producing the highest pre-weaning weights and ADG [71]. Such findings underscore the nuanced interplay of timing, nutrient type, and maternal body condition in shaping calf growth trajectories. The overall benefits of protein and energy supplementation for growth performance are summarized in Figure 4.

### 6.2. Immune Function and Health

Appropriate maternal nutrition fosters offspring’s immune competence and overall health in beef cattle. For example, calves from dams fed HE diets during late gestation had higher levels of interleukin-2 and -4, showing stronger neonatal immunity. They also had greater antioxidant capacity and superoxide dismutase activity [138]. Methionine supplementation improved calf immune responses during rearing, even though birth weights were unchanged. This suggests that methionine supports immune priming and metabolic programming more than fetal growth [66].

Trace mineral supplementation also contributes to healthier calves. Calves from dams supplemented with AAC had fewer cases of BRD and heavier weaning and slaughter weights, showing a link between immune function and growth [145]. Stephenson, et al. [146] reported that supplementing cows with 133% of NASEM-recommended copper, zinc, and manganese, especially in chelated form, improved colostrum yield, mineral transfer, and calf liver mineral status. These benefits boosted antioxidant activity and potentially enhanced immune resilience. Supplementing Cu, Mn, and Zn in hydroxychloride form reduced serum haptoglobin levels, suggesting improved immune resilience at weaning [147]. Prenatal VTM supplementation improved gut microbial diversity, reduced pro-inflammatory cytokines, and increased IP-10 levels, promoting immune priming more than birth weight gains [139]. Self-fed RUP supplementation lowered respiratory disease rates and feedlot mortality [148]. HFAT feeding also improved immune status, raising antibody titers and reducing cortisol levels in calves [71]. These effects of micronutrient supplementation on immune health are summarized in Figure 4.

### 6.3. Reproductive Development

Maternal nutrition during gestation also shapes reproductive efficiency in offspring. Post-weaning feedlot supplementation accelerates puberty in heifers. More heifers reached puberty by 18 months compared to those on pasture-only diets, likely due to increased gonadotropin secretion [76,149]. Bulls from FP-supplemented dams showed a trend toward more sperm defects, possibly due to excess scrotal fat and impaired thermoregulation [65].

Targeted protein supplementation in late gestation raises pregnancy rates and increases the number of heifers calving in the first 21 days. This suggests improved fetal reproductive and endocrine development [12,150]. However, regional forage conditions may limit effects. For example, cows grazing on endophyte-infected fescue showed no improvement in offspring reproduction, even with supplementation [151]. These reproductive outcomes are summarized in Figure 4.

Micronutrients also affect puberty onset. Heifers born to AAC-supplemented dams reached puberty sooner than those from non-supplemented groups [145]. These findings highlight the long-term value of trace mineral supplementation during gestation. By supporting endocrine and gonadal development, maternal nutrition can significantly affect fertility and lifetime productivity in the next generation of beef cattle.

### 6.4. Carcass Traits and Meat Quality

Maternal nutrition during gestation can strongly influence carcass traits and final meat quality in progeny [23,24]. Protein supplementation is often linked to better marbling scores and a higher percentage of carcasses graded USDA Choice or above. This reflects fetal programming that enhances intramuscular fat deposition [152]. These effects are summarized in Figure 4.

However, results vary: some studies report no significant effect on marbling, even when carcass weight rises [144], or minimal changes in the ribeye area and tenderness [153].

In other scenarios, MO has been shown to increase intramuscular fat, contributing to enhanced marbling in some cases. As explained in Section 5.3, overfeeding can also activate genes related to collagen formation. If collagen persists after birth, it may decrease meat tenderness. Thus, the net effect of MO on meat quality depends on balancing favorable fat infiltration against potential collagen cross-linking [13,20,23].

Micronutrient supplementation further influences carcass outcomes. For example, vitamin A during late gestation may heighten intramuscular fat deposition but can coincide with reduced tenderness if dosing is not carefully managed [127]. Meanwhile, RPF supplementation can lower overall growth rates but need not diminish tenderness or cooking losses. Maternal supplementation with different trace mineral sources (hydroxychloride, sulfate-based, organic) did not significantly influence offspring carcass characteristics, including marbling and ribeye area, indicating limited effects of mineral source on meat quality traits [147]. Producers aiming for premium carcass attributes may thus choose to tailor maternal diets for moderate muscle and marbling gains while preventing the overfeeding scenarios (i.e., >110% of requirements) that risk excessive collagen buildup.

### 6.5. Metabolic and Physiological Development

Maternal diets also influence the metabolic and physiological development of calves. For example, supplementation during mid-to-late gestation promotes gastrointestinal development. It increases rumen papillae length and villi size, which enhances nutrient absorption and structural growth [61]. RPM supplementation near conception helps steer nutrients toward muscle rather than fat, fostering steady gain through the rearing phase [66].

Additionally, metabolomic profiles in dams and calves shift in response to enhanced gestational feeding, especially for amino acid metabolism and energy pathways. Research suggests that protein–energy supplementation can raise levels of carnosine and alanine in offspring, while OCM supplementation elevates maternal folate and vitamin B12, improving fetal nutrient transfer and long-term metabolic health [55,154]. Early vitamin–mineral supplementation can likewise bolster antioxidant capacity and immune function, emphasizing metabolic resilience over immediate growth [70,82].

Late-gestation methionine supplementation specifically increases fetal growth and postnatal ADG, presumably by channeling nutrients more efficiently toward muscle [155]. These findings show that maternal nutrition not only affects birth weight but also shapes long-term metabolism, health, and growth in offspring. These metabolic improvements are summarized in Figure 4.

### 6.6. Influence of Fetal Sex on MO Outcomes

MO programs male and female offspring in markedly different ways. In female progeny, Nellore heifers born to dams under no, late-gestation (PELT), or whole-gestation (PEWG) protein–energy supplementation showed only modest early weight-trajectory differences and ultimately converged in average daily gain, ultrasound traits (ribeye area, backfat, rump fat), and age at puberty; genome-wide scans detected a single treatment-specific SNP per group, indicating minimal long-term divergence [123]. Late-gestation protein supplementation further improved heifer weaning weight, pregnancy rate, and calving age [12], whereas, in well-managed systems, varying maternal protein had no effect on heifer growth or reproduction [151]. By contrast, male calves exhibit deeper, often “silent” molecular programming despite similar growth metrics. Male progeny show greater weaning-weight sensitivity under undernutrition and steeper growth gains under adequate nutrition [156], and protein-supplementation × sex interactions affect digestibility, TDN intake, and rumination time; males increase nutrient use while females maintain feed efficiency under restriction [157]. Male fetuses of overfed dams express higher myogenic (MYOD1), adipogenic (PPARG, CEBPA, ZNF423), and fibrogenic (COL1A1, FN1) markers and possess more myocytes than females [158] and consistently outperform females in birth weight, ADG, and frame size regardless of late-gestation energy intake [14]. Maternal fat supplementation increases birth weight in males only [72]; first-trimester protein restriction boosts post-weaning weight in males but reduces it in females [159]; late-gestation Ca salts of soybean oil enhance steer ADG and carcass weight without affecting heifers [160], and methionine supplementation preferentially elevates male birth weight, hip width, and plasma amino acids [155]. Bulls from dams receiving full-gestation supplementation tend to show more sperm defects. They also exhibit SNP changes in insulin, lipid metabolism, androgen biosynthesis, and apoptosis pathways. These are accompanied by shifts in liver metabolites such as citrulline, sphingomyelins, and dicarboxylcarnitines during rearing, which normalize by finishing [56]. Complementing these findings, Hanwoo bull calves from mid-to-late-gestation overfed dams downregulate PPARGC1A, THBS1, and CD44 and upregulate ADCY6 and select lncRNAs in round and sirloin muscle, revealing profound transcriptomic remodeling despite unchanged growth performance [116].

These sex-stratified molecular and phenotypic footprints underscore the critical need to compare male and female offspring directly and to integrate genomics, transcriptomics, metabolomics, and epigenomics in fetal programming research, thereby enabling precision-feeding strategies optimized for both sexes.

### 6.7. Impact of Maternal Nutrition on Offspring Rumen Microbiome and Gut Flora

Maternal diet helps seed the calf gut microbiome. At birth, microbes in the hindgut originate from the umbilical cord (23.8%), placenta (15.6%), colostrum (14.4%), amniotic fluid (11.2%), and feces (10.5%) [161]. Gestational energy or supplement shifts maternal and offspring rumen taxa, Fibrobacter, Prevotella, and Ruminococcus, altering SCFA profiles (e.g., propionate) that influence fetal immunity and metabolism [162,163]. Probiotics and direct-fed microbes during gestation can similarly reshape maternal communities and, in turn, the calf’s early gut flora [164,165].

Late-gestation undernutrition reduces placental microbial diversity, whereas mineral supplementation increases fetal gut microbiota richness, implicating the placenta in vertical transfer [166,167]. Key rumen colonizers, including Fibrobacter succinogenes, Ruminococcus flavefaciens, Methanobrevibacter, and Prevotella, appear within minutes of birth in rumen fluid and meconium, priming fermentation and immune development [168,169].

Protein–energy programming enhances rumen papillae counts and alters cecal crypt depth in Nellore bulls, correlating with shifts in Bacteroidales BS11 and goblet cell numbers—evidence of microbiome–epithelium co-programming [170,171]. Vitamin–mineral supplementation during gestation increases calf rumen diversity and reduces Escherichia–Shigella [139,172], while dry-period concentrate feeding lowers enteric infections without altering colostral IgG [173]. Conversely, high-energy late-gestation diets reduce colostral IgG [174], whereas fish oil and linseed improve colostrum immunoglobulin content and early microbial establishment [175].

Protein–energy supplementation throughout gestation programs the offspring’s rumen and fecal microbiome. It enhances glycerophospholipid and PUFA pathways, in contrast to the methane- and amino acid-focused profiles in non-supplemented calves. Correlations between Saccharofermentans and malonylcarnitine support coordinated fermentation shifts [176]. In dairy calves, over 50% of meconium microbiota trace back to the maternal rumen, rich in Prevotella, Succiniclasticum, and Butyrivibrio, transmitting enhanced carbohydrate and lipid metabolism and reduced antimicrobial-resistance genes, whereas maternal contact alone yields only transient effects on the calf gut by four weeks. The authors of [177,178] found that although maternal contact influences calf oral microbiota transiently, diet and environment dominate gut microbiome maturation by four weeks of age.

These studies demonstrate that maternal nutrition programs the calf’s gut ecosystem via vertical microbial transfer, maternal microbiome modulation, and prenatal interventions. Future fetal programming research should integrate 16S rRNA sequencing, shotgun metagenomics, and metabolomics of calf rumen and gut contents to fully elucidate microbe-mediated developmental programming pathways.

### 6.8. Controversies and Breed-Specific Responses

Despite the general trends summarized above, responses to gestational supplementation are far from uniform (Table 2). For example, mid-gestation protein–energy inputs improved birth and weaning weights and enlarged muscle fibers in Nellore calves [64], whereas a comparable late-gestation protein or methionine boost produced little advantage over controls once postnatal management was standardized [69]. Likewise, raising total nutrients to ≈150% of requirements in the third trimester increased calf weight in British breeds [68,80] but not in Hanwoo newborn calves [61,135]. Also, similar energy surpluses showed no consistent benefit for calf growth or carcass traits in other trials [5,13,74,118]. The form of protein also matters: RUP feeding elevated dam BW and ADG but did not translate into better fertility [53].

Genetic background influences outcomes. Angus-based cattle tend to respond to moderate MO with increased marbling. In contrast, Hanwoo and Wagyu, already marbling-oriented, are more likely to upregulate fibrogenic genes like COL3A1 and FN1 under high energy intake, which may reduce tenderness [60,103]. Bos indicus types show still different patterns: in Nellore, full-gestation programming drove immune-related hepatic networks and rumen-papillae expansion without the pronounced marbling response seen in Angus contemporaries [55,171], and a multigenerational Zebu dataset attributes up to 53% of the variation in reproductive traits to the historical maternal environment [105].

Collectively, these contradictions highlight that timing, nutrient form, postnatal management, and breed biology interact to determine whether a given gestational strategy proves advantageous or counter-productive, underscoring the need for precision, breed-specific feeding guidelines.

## 7. OMICs Integration Reveals Metabolic Programming Under MO

Integrative multi-omics studies have shown that MO reprograms offspring metabolism at every regulatory level. In fetal liver, muscle, and cerebrum, RNA-Seq and co-expression networks revealed that restricting versus meeting 100% of maternal energy requirements rewires glycolysis, lipid metabolism, and neurotransmission pathways by day 50 of gestation [121]. When combined with metabolomic and epigenomic profiles related to RFI, efficient (low-RFI) offspring display muscle-promoting metabolites; nutrient-sensitive regulation of IGF1, PPARG, and MEF2A; and tissue-specific methylation shifts at IGF2, H19, and PCDH19 [134].

In Nelore bulls, WGCNA of liver transcriptome and metabolome from non-supplemented (NP), partially programmed (PP), and fully programmed (FP) dams revealed NP enrichment in epigenetic regulation and oxidative metabolism modules (e.g., SLC6A14, glutamate), PP signatures in amino acid and lipid metabolism (ODC1, arginine), and FP activation of immune-related networks (PTPRC, SLC12A8) [176]. Additionally, integrated analysis of plasma metabolome along with both rumen and fecal microbiome data indicated that FP calves maintain elevated glycerophospholipid and PUFA pathways, suggesting lasting shifts in membrane signaling and energy metabolism. Conversely, NP calves showed enrichment in amino acid metabolism, nitrogen cycling, and methane-related pathways. FP animals also demonstrated the activation of RNA-degradation and LPS-biosynthesis pathways, highlighting significant host–microbe regulatory interactions [176].

Early-gestation maternal gain further reprograms the fetal liver via coordinated epigenetic and transcriptional changes: moderate gain alters methylation at WNT7B and POU2F2, upregulates bta-miR-206 (targeting morphogenesis and ion-transport genes), and converges on PI3K-Akt, MAPK, Wnt, and mTOR nutrient-sensing pathways [97]. Prenatal protein–energy supplementation likewise reshapes maternal and calf plasma metabolomes, elevating glutamate, alanine, and phosphatidylcholines and enriching histidine and beta-alanine metabolism, suggesting persistent effects on membrane integrity, immune signaling, and transgenerational programming despite minimal overt phenotype [55].

Together, these OMICs-based insights demonstrate that MO remodels host and microbial metabolic networks across epigenetic marks, transcripts, proteins, and metabolites. Incorporating such integrated approaches in fetal programming research will deepen mechanistic understanding and enable precision-feeding strategies to optimize lifelong productivity in beef cattle.

## 8. Nutritional Strategies and Recommendations

Preventing MO in pregnant beef cows involves meeting fetal developmental demands without exceeding the recommended energy intake. Studies consistently show that feeding beyond ~110–115% of NRC guidelines in late gestation can raise intramuscular and subcutaneous fat deposition, alongside elevated collagen expression, thus compromising offspring feed efficiency and meat tenderness [13,14,118]. Consequently, producers should aim to keep maternal BCS near 5.0–5.5 at calving, reducing the risk of metabolic disorders and difficult births associated with overconditioned cows.

Mid-gestation is especially critical for fetal muscle fiber development yet allows moderate weight gains if dams are under their target BCS [3,120,142]. During this period, supplementation should largely address protein shortfalls while holding TDN around 100–110% of NRC recommendations. For example, if forage quality is marginal, offering a protein supplement at 0.2–0.3% of cow body weight can avert undernutrition without drifting into overfeeding. Monitoring forage availability, adjusting rations weekly, and periodically scoring BCS all help maintain the balance between adequate fetal nutrition and preventing MO. Moreover, nutritional strategies should account for sex-specific differences in fetal programming. Male calves exhibit pronounced sensitivity to nutritional variations, displaying deeper molecular programming with significant muscle and adipose development changes [14,155,156]. Females, in contrast, often present more subtle, yet important, growth and reproductive responses, highlighting the need for targeted nutritional management to optimize outcomes in both sexes [12,123,157].

By late gestation, fetal nutrient demands escalate. Yet, surpassing ~110–115% TDN in this phase can push cows into overconditioning. If BCS is already ≥5.5, producers should moderate any energy-dense feeds (e.g., high-corn diets) and instead rely on forage-based or moderate-concentrate rations aligned with TDN targets. Where additional protein is indicated, focusing on RUP or rumen-protected methionine supports fetal muscle growth without excessively boosting maternal fat [66,155]. Stabilizing or gradually increasing BCS if cows are <5.0 is the goal; however, pushing them above 5.5 fosters detrimental adiposity.

Micronutrient supplementation remains essential to strengthen fetal immune and antioxidant systems, yet these interventions generally do not risk MO. Because minerals and vitamins carry negligible caloric content, modest surpluses rarely spur overconditioning [70,145]. For instance, Se, zinc, copper, and manganese can be given at or slightly above recommended levels to support offspring’s immune function. Recent data suggest that hydroxychloride trace minerals (zinc, copper, and manganese) may slightly enhance calf immunity; however, carcass traits, including marbling and ribeye area, were unaffected by mineral source, emphasizing balanced mineral nutrition as key regardless of supplementation form [147]. The careful use of vitamin A in late gestation may increase intramuscular fat, though high doses could raise collagen deposition if not monitored [127]. By contrast, vitamin D aids fetal bone and muscle development, reinforcing skeletal strength without contributing extra dietary energy.

Emerging evidence also underscores the benefits of managing maternal diets to positively influence offspring gut microbiota. Including probiotics or direct-fed microbial supplements during gestation can reshape maternal rumen communities, promoting beneficial gut flora in calves, thereby enhancing early immune and metabolic development [162,164,165,171,176].

A central consideration is cost–benefit analysis. Producers must weigh whether the expense of additional feed or specialized supplements realistically translates to improvements in calf growth or carcass returns. Evidence suggests that overshooting nutrient requirements does not always yield proportional performance gains, potentially undermining profitability [89]. Thus, precision feeding through total mixed rations or targeted supplementation helps producers avoid inadvertently exceeding energy needs and incurring feed costs that fail to pay off in calf performance.

Practical precision approaches, including electronic feeders, let managers adapt daily intakes based on real-time BCS measurements, preventing cows from drifting into overconditioning late in gestation. Such dynamic fine-tuning is especially valuable if animals are predisposed (genetically or otherwise) to accumulate fat rapidly.

Recognizing breed-specific nutritional responses is critical. Angus cattle may benefit from moderate MO through enhanced marbling [75,124]. Conversely, breeds genetically predisposed to marbling such as Hanwoo and Wagyu risk increased fibrogenic gene expression (e.g., COL3A1, FN1) and decreased meat tenderness under similar nutritional conditions, thus necessitating careful dietary adjustments [60,103,116].

Finally, leveraging advanced OMICs tools (genomics, transcriptomics, and metabolomics) can refine precision feeding practices. These technologies help identify optimal nutritional interventions based on specific metabolic, genetic, and microbiome profiles, enhancing productivity and economic returns [55,97,121,134].

## 9. Conclusions

Maternal nutrition during gestation exerts a profound influence on fetal programming, postnatal performance, and sustainability in beef production. In particular, MO feeding above ~110–115% of the recommended energy can impair mitochondrial function, shift muscle development toward excess fat and collagen deposition, and diminish carcass quality. Epigenetic mechanisms such as DNA methylation, histone modifications, and microRNA regulation underlie these nutrient-driven changes by modulating fetal gene expression in muscle, adipose, and immune tissues.

Nevertheless, targeted supplementation including micronutrients (Se, zinc, copper) and amino acids (e.g., methionine), can reinforce antioxidant defenses, immune responses, and reproductive outcomes without risking overconditioning when applied judiciously. Integrating precision feeding strategies that match nutrient levels to each gestational phase, along with regular BCS, remains central to controlling MO. This approach ensures healthy fetal development, maximizes postnatal growth and carcass merit, and improves economic returns in beef operations.

Recent research underscores critical considerations, such as sex-specific programming differences, the significant impact of maternal nutrition on offspring rumen microbiota, and the varied nutritional responses among cattle breeds. These findings highlight the necessity for targeted nutritional management that considers offspring sex, microbiome modulation strategies, and breed-specific dietary recommendations to enhance overall effectiveness.

Future research should further dissect breed-specific vulnerabilities to overfeeding and the multigenerational impacts of maternal diets, particularly through advanced epigenetic profiling. Refined feeding strategies that incorporate real-time energy monitoring and cost–benefit analyses will help producers balance productivity, animal welfare, and long-term sustainability under evolving market and environmental pressures.

## Figures and Tables

**Table 2:** Conflicting outcomes of major gestational nutrition strategies and the moderating role of breed genetics in beef-cattle fetal programming studies.

Topic	Positive (Benefit Reported)	Neutral/Negative (no Benefit or Opposite)	Possible Reason(s)	Breed(s) Concerned
**Late-gestation protein + energy supplementation**	↑ birth and weaning BW, heavier carcasses (150% req.; [68,80])	No change in pregnancy rate despite ↑ BCS [59,61,67] Similar calf BW and carcass traits vs. control [5,13,61,74,118]	Gestational timing, dam BCS, post-weaning management	Multiple Bos taurus breeds; Hanwoo shows no benefit
**Mid-gestation protein supplementation**	↑ birth and weaning BW, larger muscle fibers [64]	Costa et al. [142]: mid-gestation restriction ↓ muscle fibers even at 450 d; Lawson [69]: added protein/methionine late G → minimal early-life effects	Level (restriction vs. surplus); genetic line	Angus-cross, Nellore, Wagyu
**RUP**	↑ ADG and final BW of dams [54]	No definitive fertility gain [54]	Source of protein vs. energy balance	Angus × Hereford cows
**RPM (periconception)**	↑ calf BW, ↑ post-weaning ADG [66]	Did **not** improve pregnancy outcomes in dams	May target fetal metabolism more than dam fertility	Angus × Simmental
**HE late gestation diets**	↑ calf birth BW, ↑ neonatal immune/antioxidant markers [138]	HE diet may ↓ colostral IgG [174]; other HE trials → no long-term growth advantage [13,68]	Excess energy depresses IgG; breed tolerance	Bos taurus beef breeds
**PUFA supplementation (late gestation)**	↑ steers’ ADG and HCW [160]	No response in heifer contemporaries [160]	Sex-specific adipogenic programming	Angus/Angus-cross
**Trace-mineral (Zn, Cu, Mn) supplementation**	Chelated form ↓ haptoglobin, ↑ colostrum yield and calf immunity [146,147]	No effect on marbling or REA regardless of mineral source [147]	Immune vs. carcass pathways distinct	Red Angus, Angus, Hereford mixes
**Breed-specific MO response**	Angus, marbling ↑ with moderate MO, tenderness mostly retained [75] and greater intramuscular fat deposition [124]	Hanwoo and Wagyu—already high marbling; MO ↑ collagen genes (COL3A1, FN1) → ↓ tenderness [12,116]	Genetic propensity for fat vs. collagen synthesis	Hanwoo, Wagyu, Angus, Angus × Simmental
**Bos indicus vs. Bos taurus**	Nellore bulls: full programming ↑ immune-related hepatic networks (PTPRC, SLC12A8) and ↑ papillae count in rumen [55,171]	Zebu multigenerational study: historic gestational environment explains up to 52.9% variance in repro traits [105] but growth responses less pronounced than in Bos taurus trials	Differences in placental efficiency, heat tolerance, fat partitioning	Nellore, Zebu

BW, body weight; BCS, body-condition score; ADG, average daily gain; HCW, hot-carcass weight; REA, rib-eye area; RUP, rumen-undegradable protein; HE, high-energy; PUFA, poly-unsaturated fatty acid; IgG, immunoglobulin G; MO, maternal overnutrition; d, day(s); G, gestation, ↑/↓; increase/decrease, →; leads to

## Data Availability

No new data were generated or analyzed in this study.

## Abbreviations

The following abbreviations are used in this manuscript:

AA	Amino acid
AAC	Organic-complexed trace minerals
ADCY6	Adenylyl cyclase 6
ADG	Average daily gain
AGR	Agouti-related peptide
AMPK	AMP-activated protein kinase
APC	Adenomatous polyposis coli (Wnt co-factor)
BAT	Brown adipose tissue
BCS	Body-condition score
BRD	Bovine respiratory disease
BW	Body weight
CBS	Cystathionine β-synthase
CP	Crude protein
CPT2	Carnitine-palmitoyl-transferase 2
DMI	Dry-matter intake
ECM	Extracellular matrix
FASN	Fatty-acid synthase
FCR	Feed-conversion ratio
FGF2	Fibroblast growth factor 2
FN1	Fibronectin 1
FOXO1/3	Forkhead box O1/O3
HCW	Hot-carcass weight
HE	High-energy (diet)
HFAT	High-fat bakery-waste diet
HPN	High plane of nutrition
HSD	High-starch diet
IgG	Immunoglobulin G
IUGR	Intrauterine growth retardation
JAK-STAT	Janus-kinase/signal-transducer-and-activator-of-transcription pathway
lncRNA	Long non-coding RNA
LFAT	Low-fat bakery-waste diet
LOX	Lysyl oxidase
LPN	Low plane of nutrition
ME	Metabolizable energy
miRNA	Micro-RNA
MO	Maternal overnutrition
mTOR	Mechanistic target of rapamycin
MPN	Medium plane of nutrition
NRC	National Research Council
OCM	One-carbon metabolites
PUFA	Poly-unsaturated fatty acid
RFI	Residual feed intake
RPF	Rumen-protected fat
RPM	Rumen-protected methionine
RUP	Rumen-undegradable protein
SCFA	Short-chain fatty acid
Se	Selenium
TDN	Total digestible nutrients
VTM	Vitamin–mineral supplement
WAT	White adipose tissue
WGCNA	Weighted gene-co-expression network analysis
ZFP423	Zinc-finger protein 423
